# Reducing trauma-related parental stress in neurodevelopmental disorders: a randomized feasibility study

**DOI:** 10.1007/s00431-026-06848-z

**Published:** 2026-03-15

**Authors:** Cesare Cavalera, Alessia Incerti, Isabel Fernandez, Ignacio Jarero, Nicolle Mainthow, Francesco Pagnini

**Affiliations:** 1 Department of Psychology, Università Cattolica del Sacro Cuore, Via Nirone 15, Milan, 20123 Italy; 2 Centro Di Ricerca e Studi in Psicotraumatologia, CRSP, Milan, Italy; 3 Research Department, EMDR Mexico National Association, Mexico City, Mexico

**Keywords:** Neurodevelopmental disorders, Family resilience, Traumatic symptoms, EMDR-IGTP-OTS, Randomized feasibility trial

## Abstract

This study aimed to explore the feasibility of the Eye Movement Desensitization Reprocessing Integrative Group Treatment for Ongoing Traumatic Stress (EMDR-IGTP-OTS) focused on reducing traumatic stress symptoms in parents of children with neurodevelopmental disorders (NDDs). A process-outcome study was conducted, recruiting parents with children diagnosed with NDDs through the collaboration of NDDs family associations in Emilia-Romagna, Italy. 34 Parents experiencing ongoing traumatic symptoms were randomly assigned to the EMDR group intervention (*n* = 19) or to the control condition (*n* = 15). Participants were assessed at pre-test, post-test, and 3-month follow-up. Data were gathered through structured questionnaires screening psychological traumatic stress symptoms. Qualitative data were collected through a therapeutic process diary filled out by the participants. Statistical analysis included descriptive statistics, independent sample t-test, non-parametric analysis, and multiple regression. Mann–Whitney test showed significant differences in traumatic stress symptomatology reduction between the groups, Wilcoxon test showed significant reduction in traumatic stress symptoms over time, and multiple regression data showed that the number of sessions attended predicted the intensity of the traumatic stress symptoms reduction. Qualitative thematic analysis showed that the intervention proved to be feasible and resulted in an increase in positive aspects (e.g., body connection, agency, and pleasant emotions) as the intervention progressed.

*Conclusion*: The EMDR-IGTP-OTS is a promising intervention for reducing trauma related parental stress symptoms in parents of children with NDDs. Its high feasibility makes it a suitable option for integration into clinical practice to support families facing trauma-related parenting challenges.

**Table:** 

**What is Known:** • *Parents of children with neurodevelopmental disorders experience high levels of chronic stress and trauma-relatedsymptoms, yet evidence-based psychological interventions specifically targeting caregivers remain limited.*
**What is New:** • *This pilot randomized feasibility study provides preliminary evidence that the EMDR-IGTP-OTS group protocol is feasible and may reduce trauma-related stress symptoms in parents of children with neurodevelopmental disorders.*

**Supplementary Information:**

The online version contains supplementary material available at 10.1007/s00431-026-06848-z.

## Introduction

Parents of children with neurodevelopmental disorders (NDDs) are at greater risk for increased stress, usually comparable to those associated with traumatic events [1]. While NDDs encompass a broad and heterogeneous range of diagnoses, research on specific subgroups, such as parents of children with autism spectrum disorder or intellectual disabilities, suggests that these symptoms can emerge shortly after the child’s diagnosis and may intensify over time due to the burden of continuous caregiving [2]. Increasingly, the shift from hospital-based to home-based care has positioned parents as primary caregivers and central members of healthcare teams [3, 4]. This prolonged exposure to chronic distress may lead to cumulative trauma, placing caregivers at a higher risk of developing repeated post-traumatic stress symptoms compared to individuals exposed to a single traumatic event [5]. Within this framework, parents, particularly those dealing with severe behavioral challenges in specific NDD subtypes, may become “secondary victims” of their child’s condition, with elevated distress levels that could undermine their ability to provide effective care, thereby exerting a significant impact on the stress experienced by their children with NDDs [6].

Parents of children with various neurodevelopmental disorders face a greater degree of psychological distress than parents of typically developing showing more anxiety and burnout and may more engage in self-harming or suicidal behaviors [7, 8].

Given the great need for support intervention and its proven effectiveness in treating post-traumatic stress symptoms, Eye Movement Desensitization and Reprocessing (EMDR) therapy may be a suitable option for this population [9]. Specifically, the EMDR Integrative Group Treatment Protocol for Ongoing Traumatic Stress (EMDR-IGTP-OTS) has advantages over other group interventions in terms of time efficiency, resource allocation, and treatment efficacy [9–12]. Despite these promising findings, research on the EMDR-IGTP-OTS with parents of children with NDDs remains limited. Incerti and colleagues [13] implemented a combined EMDR-IGTP and cognitive behavioral therapy intervention for parents of children with disabilities, reporting a post-traumatic growth. Nevertheless, the combined treatment limits the ability to isolate the EMDR-IGTP’s effectiveness, and the intervention primarily targeted trauma related to the diagnosis itself, neglecting other caregiving stressors that accumulate over time. Passoni and colleagues (5) evaluated the EMDR-IGTP in caregivers of individuals with dementia, finding reductions in trauma, anxiety, and depression. However, caregivers of adult patients differ markedly from parents of minors with NDDs in terms of autonomy, emotional load, and educational responsibility. Therefore, additional research is needed to assess whether the EMDR-IGTP-OTS is both acceptable and effective for this specific caregiver population, and whether it can enhance psychological well-being and reduce cumulative traumatic stress symptoms. To address this gap, the present study primarily evaluates the feasibility and preliminary effectiveness of the EMDR-IGTP-OTS in alleviating trauma-related parental stress among parents of children with NDDs, who are exposed to prolonged and repeated stressful events.

We hypothesize that the intervention will demonstrate high feasibility, as indicated by participant adherence to the sessions. Regarding effectiveness, we expect the experimental group to show a significant reduction in the traumatic stress symptoms compared to the control group, with these improvements maintained at the 3-month follow-up. We also hypothesize that the narrative entries will reveal a transition from predominantly trauma-related challenges to the emergence of adaptive resources, reflecting the reprocessing steps of the EMDR-IGTP-OTS protocol.

## Methods

### Study design

This feasibility study employed a randomized controlled design with three assessment times at pre-test, post-test, and 3-month follow-up. Data were collected by the research team and registered via Qualtrics software. The research team was responsible for both participant recruitment and data collection, which took place from September 2024 to March 2025. The study design was registered on PsyArXiv. All participants provided informed consent, and ethical approval was granted by the ethical committee of Università Cattolica del Sacro Cuore (protocol n° 156/24).

### Study participants

The study population included 34 parents of children under 18 years old with clinically diagnosed NDDs. The inclusion criteria for parents were (1) being the parent of a minor child diagnosed with NDDs (according to the DSM V criteria); (2) scoring 33 or higher on the Impact of Event Scale – Revised (IES-R); and (3) providing informed consent for study participation. The exclusion criteria were (1) pregnancy; (2) diagnosed psychiatric disorder; (3) suicidal ideation; (4) self-harming behaviors; (5) intellectual disability and/or significant cognitive impairment; (6) severe difficulties in understanding or expressing in Italian; (7) ongoing psychotherapeutic therapy; and (8) psychiatric intervention change in the last 3 months. The majority of children had a primary diagnosis of autism spectrum disorder (*n* = 32, 94.1%), while one child had a diagnosis of ADHD and one a diagnosis of Specific Learning Disorder. More information about diagnosis, comorbidities and clinical characteristics of the sample are reported in Table S1. Diagnoses were established by specialized public child neuropsychiatric services within the Italian National Health System, including the Local Health Authority of Romagna and the IRCCS Fondazione Stella Maris (Pisa, Italy).

### Sample size

A priori power analysis was conducted based on the effect size reported by Encinas et al. [4], who observed a large between-group effect (*ηp*^2^ = 0.223). An effect of this magnitude corresponds approximately to *f* = 0.60, for which groups of about 15 participants typically provide high statistical power in repeated-measures designs. We therefore set a target recruitment of 40 participants, allowing for potential attrition while ensuring adequate numbers to explore feasibility indicators and detect preliminary signals of efficacy. The final sample of 34 parents reflects the number of eligible participants recruited during the study period and remains consistent with methodological recommendations for feasibility trials.

### Procedure

Participants were recruited through a snowball sampling method with the collaboration of family associations of children with NDDs in the Emilia Romagna region (Italy). After expressing interest, potential participants were contacted by a research team member, who provided detailed information about the study’s aims, content, procedure, and participation requirements. This interaction ensured that participants had sufficient opportunity to ask questions and address any concerns. Participants were asked to complete the IES-R referring to the most stressful event related to their parenting experience with their child with NDDs. In the case of a score of 33 or higher on the IES-R scale, which indicates the presence of traumatic stress symptoms [15], the participant was informed about the possibility of participating in the study. Psychological assessments were administered by a trained researcher from the research team who was blinded to the group assignment.

Of the 54 parents initially assessed, 20 were excluded because they did not meet the inclusion criteria. The remaining 34 parents were then randomly assigned to the EMDR-IGTP-OTS treatment group (*n* = 19) or to a control waitlist group (*n* = 15). The allocation was performed using a simple randomization procedure based on a computer-generated sequence to ensure concealment and eliminate selection bias. To verify the success of the randomization, the groups were compared at baseline, showing no significant differences in demographic or clinical variables (Table 1). The eight phases of the EMDR-IGTP-IOTS were implemented and applied by conducting two sessions per day for three distinct and consecutive days [10]. The EMDR-IGTP-OTS protocol incorporates the 8 phases of the EMDR therapy standard protocol to provide individual EMDR therapy in a group format. One therapist who was trained in the EMDR-IGTP-OTS led the sessions, while two psychotherapists served as Emotional Protection Team members and available for support. The protocol utilizes the butterfly hug (BH) as self-administered bilateral stimulation and elements of art therapy to facilitate the full reprocessing of the pathogenic memory network associated with the stressful events. As indicated by EMDR-IGTP-OTS procedures, to facilitate the reprocessing without the need for verbal disclosure within the group, participants were provided with blank sheets and wax crayons. Under the guidance of the therapist, they were directed to represent their internal experiences and emotional shifts through drawings, strategically alternating these art-therapy moments with sets of BH as bilateral stimulation. Reprocessing focused on the stressful parenting events and started with the most distressing one (for which the IES-R was administered). In cases where all target memories were reprocessed before the end of the 6 sessions, the remaining time was used for stabilization and reinforcement of the positive results achieved, focusing on the future template and adaptive resources.

**Table 1:** Descriptive and clinical characteristics of the participants

	EMDR group condition (1) *N* = 19	Control condition (2) *N* = 15	Total sample *N* = 34	Between group comparison (*p*)
Gender				0.436
Male	3	4	7	
Female	16	11	27	
Age	45.32 (4.97)	45.27 (6.41)	45.29 (5.56)	0.972
Years from diagnosis	5.40	7.37	6.50	0.105
Educational status				0.369
Basic education	8	8	16	
Higher education	11	7	18	
Marital status				0.432
Married	10	12	22	
Separated/divorced	6	2	4	
Single	3	1	4	
STAI-T	28.37 (8.86)	24.67 (3.96)	26.74 (25)	0.158
IES-R T0	46.95 (12.27)	44.60 (9.26)	45.91 (10.95)	0.676
Δ IES-R_T1-T0_	− 35.68 (14.39)	− 16.60 (15)	− 30.68 (17.22)	**0.002**
Δ IES-R_T2-T0_	− 35.39 (16.63)	− 22.20 (15.58)	− 30 (17.35)	**0.044**
SUD T0	7.95 (8)	7.67 (9)	7.82 (1.99)	0.916
Δ SUD_T1-T0_	− 4.67 (3.27)	− 0.90 (1.91)	− 3.32 (2.87)	** <.001**
Δ SUD_T2-T0_	− 4.96 (2.61)	− 1.27 (1.58)	− 3.32 (3.37)	**0.010**

*p* values refer to between-group comparisons (EMDR vs control) at baseline. For categorical variables, *p* value was evaluated through *T*-test, for continuous variables *p* value was evaluated through the Mann-Whitney test.

A post-test assessment was conducted one week after the last administration of the intervention, and a follow-up assessment was conducted three months after the last administration of the intervention. At follow-up (T2), one participant from the EMDR group and four from the control group were lost to follow-up, resulting in an overall attrition rate of 14.7%. At the end of the follow-up period, all participants in the control group were offered the same treatment.

## Measures

### General information sheet

A demographic and clinical information sheet was used to collect details about the child (e.g., diagnosis, gender, age, and diagnosis age) and the parent (e.g., gender, age, educational level, and marital status information).

### Impact of Event Scale-Revised

Impact of Event Scale-Revised (IES-R) is a 22-item self-report measure that assesses subjective distress caused by traumatic events [15]. The IES-R yields a total score of Traumatic Symptoms Severity (TSS, ranging from 0 to 88), and subscale scores can also be calculated for the Intrusion, Avoidance, and Hyperarousal subscales. Scores greater than or equal to 33 indicate the presence of traumatic stress symptoms [16].

### Subjective Unit of Disturbance Scale

The Subjective Unit of Disturbance (SUD) scale was used to assess the level of disturbance of the traumatic stress experiences [17]. Participants were asked to rate their level of disturbance related to their most stressful parenting experience event from 0 to 10, where 0 shows no disturbance and 10 shows maximum disturbance.

### Therapeutic process diary

After each of the three daily sessions, participants were asked to fill out a diary to collect therapeutic process information (free feedback, new things learned after the session, most positive and negative aspects).

### Statistical analyses

Quantitative data analyses were performed using *Jamovi* software. Descriptive statistics were calculated to summarize sample characteristics and main variables and are reported in Table 1. For categorical variables, differences were evaluated through a *T*-test. For continuous variables, since data were not normally distributed, Mann–Whitney tests were performed to compare Δ IES-R and Δ SUD between the two group conditions. Changes in the IES-R scores were considered the primary outcome measure, whereas the SUD score was intended to provide secondary, exploratory insights into the participants’ perceived distress levels.

Within-group changes over time (pre-test, post-test, and 3-month follow-up) were examined using Wilcoxon tests. Since not all the participants in the treatment group attended all six sessions, a linear regression analysis was performed to investigate whether the number of EMDR-IGTP-OTS sessions (N° of sessions) attended by the participants, significantly predicted the reduction in traumatic stress symptoms. To explore potential confounding effects, preliminary non-parametric analyses were performed. Spearman’s correlations were used to examine associations between years since diagnosis and changes in IES-R scores. Additionally, Kruskal–Wallis tests were performed to assess the impact of marital status, educational level, and child’s diagnosis on the same outcome measures, while Mann–Whitney U tests were used for “sex” variable. Variables that showed a significant association with the outcomes in these preliminary tests were subsequently included as covariates in the multiple regression models to control for their potential confounding effects. Effect sizes (partial eta-squared for ANOVA, and *R*^2^ for regression) were reported to assess the magnitude of the observed effects. A post-hoc power analysis was conducted for the primary outcome (ΔIES-R T1–T0 between groups) using G*Power 3.1, based on the observed effect size, alpha = 0.05, and the actual sample size. All analyses were conducted with a significance level set at *p* < 0.05.

Qualitative thematic analysis was conducted using *Ligre* software (v. 6.5.1). The analyses involved a first extrapolation of the emerging sub-categories of the Therapeutic Process Diary that described the different aspects of the participants’ feedback after the session. Subsequently, sub-categories were grouped into superordinate conceptual categories, and frequency counts were calculated. Participant satisfaction was also assessed at each diary completion using a 0–10 Likert scale rating perceived usefulness of the intervention.

## Results

The descriptive statistics for all the main study variables and the sociodemographic characteristics of the sample are reported respectively in Table 1. No significant differences were found between groups in terms of gender, age, marital status, or educational status distribution or child’s diagnosis. Significant differences in the frequency distribution of stressor categories were examined using a chi-square test, A chi-square goodness-of-fit test indicated that stressor categories were not equally distributed, (*χ*^2^ = 11.88, *p* = 0.008), with behavioral management in public settings being the most frequently reported stressor.

Mann-Whitney tests revealed significant differences between the two groups considering both Δ IES-R_T1-T0_ (U = 53, *p* < 0.005, *r* = 0.63) and Δ IES-R_T2-T0_ (U = 47.52, *p* < 0.05, *r* = 0.47) and Δ SUD _T1-T0_ (U = 35, *p* < 0.001, *r* = 0.75) and Δ SUD _T2-T0_ (U = 36.50, *p* < 0.05, *r* = 0.59). The treatment group showed a greater reduction compared to the control group in both of Δ IES-R and Δ SUD scores in the different times. The post-hoc power analysis indicated a statistical power of 0.99, suggesting that the study was adequately powered to detect the observed effects and minimizing the risk of a type II error, despite the exploratory nature of the study. Wilcoxon test revealed that both groups showed a significant reduction in Δ IES-R and Δ SUD scores from pre- to post-treatment, with larger effect sizes observed in the treatment group (W = 190, *p* < 0.001 d = 1) than in the control group (W = 112, *p* < 0.005 d = 0.87). Similarly, Wilcoxon test showed significant reduction in Δ IES-R and Δ SUD scores from pre-treatment to follow-up (treatment group: W = 171, *p* < 0.001 d = 1; control group: W = 53, *p* < 0.05 d = 0.93).

Analyses exploring potential confounding and moderating factors showed that years since diagnosis was significantly correlated with ΔIES-R T2–T0 (Spearman’s *ρ* = 0.40, *p* = 0.031). No significant associations emerged between years since diagnosis and the other outcome variables (all *p* > 0.05). Additional exploratory analyses examining demographic and clinical variables (age, sex, child’s diagnosis, marital status, and educational level) did not reveal significant correlations or group differences in relation to changes in IES-R or SUD scores (all *p* > 0.05). Based on these findings, years since diagnosis was included as a covariate only in the regression model predicting ΔIES-R T2–T0. For all other outcomes, more parsimonious models without covariates were estimated. Analysis of the distribution of stressful events revealed that the categories were not equally represented among participants (*χ*^2^ = 11.88, *p* < 0.05).

Linear regressions were conducted using respectively Δ IES-R_T1-T0_ (*F*_(1, 32)_ = 7.89, *p* < 0.01, *R*^2^ = 0.20), Δ SUD_T1-T0_ (*F*_(1, 32)_ = 21.15, *p* < 0.001, *R*^2^ = 0.40), and Δ SUD_T2-T0_ (*F*_(1, 26)_ = 10.14, *p* < 0.005, *R*^2^ = 0.28) as dependent variables, with the number of attended sessions as independent variable. As shown in Table 2, N° of attended sessions significantly predicted Δ IES-R_T1-T0_, Δ IES-R_T2-T0_, Δ SUD_T1-T0_, and Δ SUD_T2-T0_. Additionally, a multiple linear regression was performed for Δ IES-R_T2-T0_ (*F*_(2, 26)_ = 8.28, *p* < 0.05, *R*^2^ = 0.39), including both the number of sessions and the years since diagnosis as independent variables. The overall model was significant, and number of sessions remained a significant predictor after controlling for years since diagnosis.

**Table 2:** Regression analysis on Δ IES-R_T1-T0_ and Δ IES-R_T2-T1_

Dependent variable	Independent variable	B	*t*	*p* value
Δ IES-R_T1-T0_	N° of sessions	− 2.78	− 2.81	** <.01**
Δ IES-R_T2-T0_	N° of sessions	− 2.71	− 2.10	** <.01**
	Years from diagnosis	2.42	3.24	**<.005**
Δ SUD_T1-T0_	N° of sessions	−.061	− 4.60	** <.001**
Δ SUD_T2-T0_	N° of sessions	−.062	− 3.18	** <.005**

**Fig. 1:**
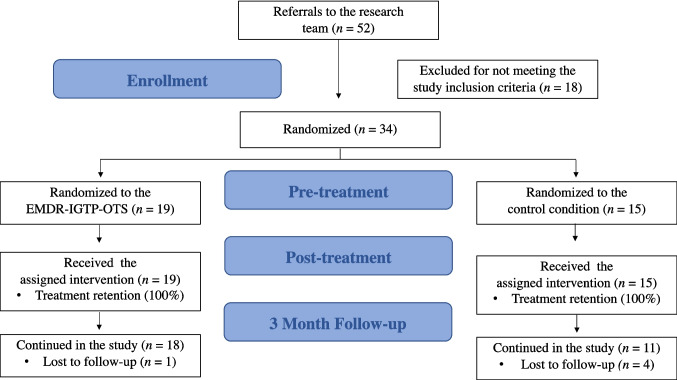
Flowchart of study participants

**Fig. 2:**
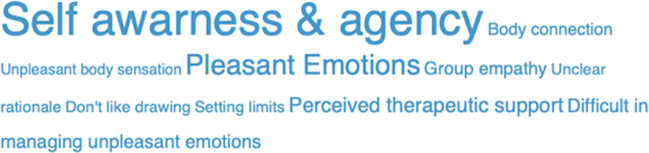
Leaf cloud of the therapeutic process diary

Participant satisfaction assessed at each diary was high, with a mean perceived usefulness rating of 7.88 (0–10 scale), supporting the acceptability of the intervention. While the lead therapist guided the group through the 8 phases, the other members of the emotion protection team monitored individual reactions to the butterfly hugs and art-therapy drawings, ensuring that any signs of over-arousal were promptly identified. No emergency situations occurred that required special intervention by the emotion protection team members. The first extrapolation of participants’ Therapeutic Process Diary identified ten sub-categories of content as reported in the Leaf Cloud (Figs. 1 and 2). Subsequently, sub-categories were grouped into two superordinate conceptual categories (pleasant and unpleasant aspects) as reported in the Codification Tree in Fig. S1.

Table 3 and Fig. S2 show the frequency of the extracted categories of participants’ Therapeutic Process Diary throughout the EMDR-IGTP-OTS intervention.

**Table 3:** Frequency of the extracted categories from therapeutic process diary

	Sessions 1 & 2	Sessions 3 & 4	Sessions 5 & 6	Tot
Pleasant aspects	65.96%	72.20%	81.13%	73.38%
Self-awareness & agency	27.66%	37.04%	45.28%	48.72%
Pleasant emotions	23.40%	16.67%	15.09%	23.93%
Group empathy	10.64%	3.70%	5.66%	8.55%
Body connection	4.26%	7.41%	3.77%	6.84%
Perceived therapeutic support	8.51%	7.41%	11.32%	11.97%
Unpleasant aspects	34.04%	27.78%	18.87%	26.62%
Setting limits	6.38%	1.85%	5.66%	18.92%
Difficult in managing unpleasant emotions	6.38%	14.81%	1.89%	32.42%
Don’t like drawing	4.26%	3.70%	5.66%	18.92%
Unclear rationale	6.38%	1.85%	3.77%	13.51%
Unpleasant body sensation	2.13%	5.56%	1.89%	16.22%

## Discussion

The present study primarily evaluated the feasibility and preliminary effectiveness of the EMDR-IGTP-OTS intervention on traumatic stress symptoms in a sample of parents of children with NDDs, a population for whom cumulative caregiving stressors often remain unaddressed in early interventions. In line with our initial hypothesis, the data suggested a satisfactory level of feasibility, as reflected by participant adherence to the protocol. Results from Mann–Whitney indicated a more significant reduction of Δ IES-R scores in the treatment group compared to the control group. These preliminary findings provide support for our hypothesis regarding the intervention’s effectiveness in alleviating symptoms in parents exposed to prolonged stressful events. Consistently with other studies, Δ IES-R significant differences were supported by large effect sizes, confirming a clinically meaningful improvement in the treatment group thanks to the intervention provided [11]. In addition to the Δ IES-R primary outcomes, Δ SUD exploratory indicator indicated a trend toward improvement that was consistent with the primary findings observed in the IES-R scores. Results from the Wilcoxon test showed a reduction of IES-R and SUD scores from pre-test to post-test and from pre-test to follow-up. As in previous studies [5, 14], these data provide evidence that the intervention enables the pathogenic memory reprocessing, moving information from a maladaptively stored state progressively to a more adaptive state over time. Notably, as demonstrated by the regression data, the greatest benefits increased with the number of sessions attended. Thus, full participation in all six sessions appears crucial for maximizing the therapeutic effects of the intervention. Results from multiple regression model suggest that parents who had been dealing with this condition for a greater number of years appeared to show a more limited reduction in traumatic stress at the follow-up. This may suggest that the chronicity of the caregiving burden may make clinical gains more difficult to sustain over time. Importantly, the number of sessions remained a robust and significant predictor of improvement (*p* = 0.006) even after adjusting for the years since diagnosis, confirming that the treatment’s efficacy persists regardless of the family’s clinical history. These findings have significant clinical implications for a comprehensive, family-centered care model. Our results suggest that the chronicity of caregiving makes clinical gains more difficult to sustain, emphasizing the need for early psychological screening immediately following a neurodevelopmental diagnosis. Increased awareness among pediatricians and mental health specialists regarding these traumatic symptoms can lead to more effective family-centered care. Furthermore, the high prevalence of distress related to behavioral management in public settings (*n* = 16) reported in Table S1 highlights that many parental stressors are “ongoing” and deeply rooted in daily life. These findings align with previous literature showing that higher levels of children’s internalizing and externalizing behavioral problems are associated with increased parental stress and with the perception of having a “difficult child” and a strained parent–child interaction [18–20]. Such behavioral and emotional challenges may contribute to persistent stress exposure in caregivers, particularly in everyday social contexts. Rather than treating parental distress as an isolated acute episode, healthcare providers and mental health professionals should implement collaborative, long-term monitoring.

Differently from Incerti and colleagues [13], the present study applied EMDR-IGTP-OTS as a stand-alone treatment explicitly addressing both initial and cumulative stressors, yielding reductions in trauma symptoms of large magnitude. These data extend Passoni and colleagues [5] findings that demonstrated EMDR-IGTP-OTS’s effectiveness in dementia caregivers, and generalize these results to parents of minors with NDDs—a group facing different emotional, educational, and autonomy-related demands.

Textual analysis revealed that the experience was feasible and well-tolerated by participants: the more pleasant aspects were linked to a sense of agency (growing mastery over accessing stressful experiences), increased self-awareness, and pleasant emotions (particularly calm and relaxation). Less pleasant experiences were linked to some difficulties in the initial group setting (comprised of people who had not previously met) and managing unpleasant emotions. As the intervention progressed, the participants’ experiences evolved: the number of positive aspects related to the experience increased, and the difficulty in reprocessing experiences related to stressful emotions peaked midway through the intervention and then decreased in the final sessions. This trajectory mirrors the non-linear but stabilizing pattern described in the Adaptive Information Processing model [17], where intense processing phases are often followed by sustained symptom relief.

The present study showed that the EMDR-IGTP-OTS represents a rapid, low-cost, and effective option for supporting parents of children with NDDs. Nevertheless, some limitations must be considered. First, the small sample size may restrict the generalizability of the findings. While the post-hoc power analysis suggested adequate power to detect the observed effects, these results should be interpreted in light of the study’s feasibility design and limited sample size.

Additionally, the reliance on self-report measures may have introduced response biases. Moreover, the study was not designed or powered to test efficacy in a full randomized controlled trial framework. The use of a waitlist control condition represents a limitation. While this design was appropriate for the aims of a feasibility study, it does not allow for controlling non-specific factors such as expectancy effects, attention from clinicians, or the potential benefits of study participation itself (e.g., Hawthorne effect). Consequently, improvements observed in the EMDR group and considered preliminary, warranting confirmation in future studies employing active control conditions. Strengthened by the feasibility insights obtained here, future research should therefore prioritize conducting a well-powered RCT to evaluate the intervention’s effectiveness more rigorously. Future studies should address these limitations by employing larger randomized samples including extended follow-up assessments, and comparing this intervention with other active trauma-focused treatments (e.g., CBT intervention or Emotion-Focused Skills Training) to determine relative efficacy [18]. Although the intervention targeted cross-diagnostic psychological processes, the specific stressors associated with different NDD conditions (e.g., symptom severity, functional levels, or comorbidities) could impact parental stress levels differently. Consequently, future studies would benefit from employing larger and more clinically homogeneous samples to investigate whether specific diagnostic profiles or functional impairments moderate the intervention’s efficacy, allowing for more tailored support strategies. Additionally, consideration should be given to other potential confounding factors such as socioeconomic status, family support or the role of trait-level factors, such as depressive predisposition or proneness to shame, that may exacerbate individuals’ responses to stressful events [21, 22]. Future research should consider incorporating the PTSD Checklist-5 (PCL-5), as it complies with the current DSM-5 and includes specific assessments for feelings of guilt, shame, and depressive mood symptoms frequently observed in parents of children with NDDs. Finally, while the SUD scale confirmed its relevance in providing useful exploratory indicator of subjective emotional distress [23, 24], the use of SUD as a longitudinal outcome measure outside of the therapeutic session requires careful interpretation compared to standardized tools. Therefore, our primary conclusions are anchored to the IES-R, and future research should prioritize standardized psychometric instruments to confirm these subjective trends.

Despite these constraints, these results suggest that the protocol could be initiated for each parent from the moment of the NDDS diagnosis, and possibly integrated with additional sessions over time.

A relevant consideration for the scalability of this intervention concerns the resource-intensive nature of the protocol. Although this type of intervention involves multiple therapists forming the EPT, their presence was considered advisable to enhance the emotional safety and monitoring of group members during the reprocessing of traumatic memories. While previous studies have primarily focused on individual EMDR interventions for parental stress related to children with NDDs or children with serious, chronic medical conditions [25, 26], our findings suggest that the EMDR-IGTP-OTS protocol may offer comparable clinical benefits within a group setting. Compared to the smaller number of participants typically reached through individual EMDR settings, the group format may allow for treating a larger number of participants simultaneously compared to traditional individual EMDR therapy. Additionally, its short duration makes it particularly suitable because, during ongoing traumatic stress conditions, where stressors are persistent and ongoing, finding extended periods for therapeutic engagement can be extremely challenging. an intervention that requires a limited time commitment may be particularly beneficial. Further, the EMDR-IGTP-OTS can help participants in coping with everyday parenting-related stressful events without new stressful experiences triggering unresolved past traumas. This can be beneficial for parents, allowing them to live a less traumatic life, and for children, who may gain more present and emotionally available caregivers, less burdened by the accumulation of stressful events in their family history. In conclusion, the present findings provide preliminary support for the feasibility and acceptability of an EMDR-based intervention for parents of children with neurodevelopmental disorders. While reductions in trauma-related distress were observed, these results should be considered exploratory and do not allow for definitive conclusions regarding clinical efficacy. Future adequately powered randomized controlled trials, including active control conditions, are needed to confirm the effectiveness of this intervention and to clarify its specific mechanisms of action.

## Supplementary Information

Below is the link to the electronic supplementary material.

ESM 1 (DOCX 276 KB)

## Data Availability

Data are available upon reasonable request to the corresponding authors.
