# Supplementary material for: Reducing trauma-related parental stress in neurodevelopmental disorders: a randomized feasibility study
Source: Eur J Pediatr. 2026 Mar 15;185(4):182. doi: 10.1007/s00431-026-06848-z (PMC12988956; doi:10.1007/s00431-026-06848-z)
Supplement: Supplementary file 1 — (DOCX 276 KB) [file 431_2026_6848_MOESM1_ESM.docx]

**Fig. S1** Codification tree of Therapeutic Process Diary


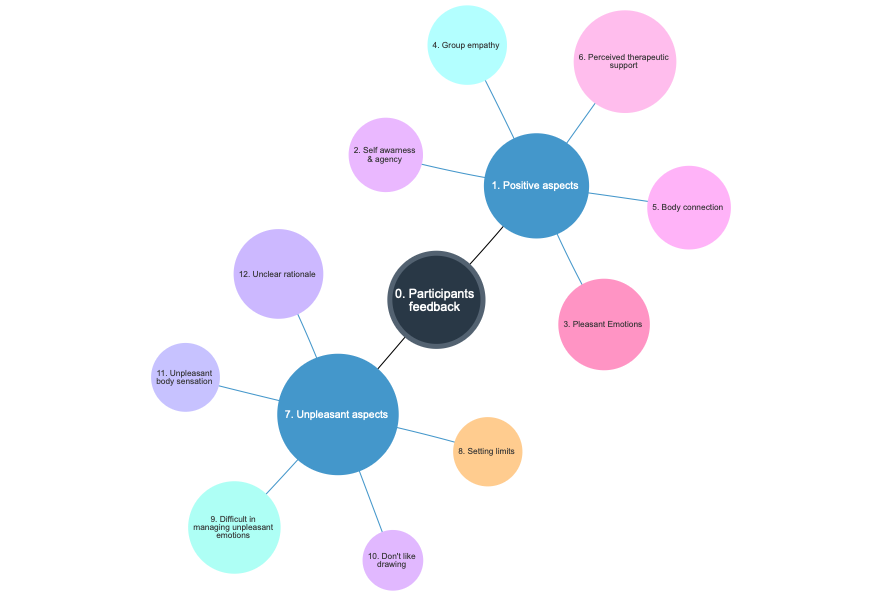


**Fig. S2a** Participants feedback throughout the therapeutic sessions

**Fig. S2b** Participants feedback throughout the therapeutic sessions

**Fig. S3** Participants feedback during the therapeutic sessions

**Table S1** Clinical characteristics of children and categories of parental stressors.

|  | N (%) |
| --- | --- |
| *Primary Diagnosis* |  |
| ASD | 32 (94.1%) |
| ADHD | 1 (2.9%) |
| SLD | 1 (2.9%) |
| *Comorbid Conditions* |  |
| Intellectual Disability | 2 (5.8%) |
| ADHD | 2 (5.8%) |
| Hypoacusia | 1 (2.9%) |
| Down Syndrome | 1 (2.9%) |
| ODD | 1 (2.9%) |
| *Parents’ Stressors* |  |
| Behavioral crisis in public settings | 16 (47.06%) |
| Diagnosis communication | 9 (26.47%) |
| Chronic caregiving burden | 7 (20.59% |
| Treatment-related decisions | 2 (5.8%) |

ASD: Autism Spectrum Disorder; ADHD: Attention Deficit Hyperactivity Disorder;

SLD: Specific Learning Disabilities; ODD: Oppositional Defiant Disorder
